# Solvothermal synthesis of pure and Sn-doped Bi_2_S_3_ and the evaluation of their photocatalytic activity on the degradation of methylene blue

**DOI:** 10.1186/s13065-021-00792-9

**Published:** 2021-12-18

**Authors:** Violet M. Nkwe, Damian C. Onwudiwe, Mayowa A. Azeez

**Affiliations:** 1 Material Science Innovation and Modelling (MaSIM) Research Focus Area, Faculty of Natural and Agricultural Sciences, North-West University (Mafikeng Campus), Private Bag X2046, Mmabatho, 2735 South Africa; 2 Department of Chemistry, Faculty of Natural and Agricultural Sciences, North-West University (Mafikeng Campus), Private Bag X2046, Mmabatho, 2735 South Africa; 3 Department of Industrial Chemistry, Ekiti State University, P.M.B 5363, Ado Ekiti, Ekiti State Nigeria

**Keywords:** Bismuth sulphide, Sn-doped, Nanorods, Photocatalysis, Methylene blue

## Abstract

**Background:**

A large volume of dye molecules finds its way into the environment, accumulates in water bodies, and makes the aquatic system unsafe to human health. Due to the complex nature of these dye materials, most of the conventional techniques are not effective for their removal. Semiconductor photocatalysis has emerged as a promising technique for  the destruction of organic pollutants under UV or visible light irradiation. Among the semiconductors, Bi_2_S_3_ is widely employed in photocatalysis due to its non-toxicity and chemical stability. However, one of its problems is the high recombination rate of the charge, and various methods have been employed to enhance the photo-reactivity. One of  these methods is the incorporation of transition elements.

**Results:**

Herein, a facile solvothermal method was used to prepare Bi_2_S_3_ nanorods and needle- shaped Sn doped Bi_2_S_3_, using bismuth(III) tris(*N*-phenyldithiocarbamate) as a single-source precursor. The prepared nanomaterials were characterized, and used as efficient photocatalyst for the photo enhanced degradation of methylene blue (MB) dye under visible light irradiation. The nanomaterials exhibited very good photocatalytic activity towards the photo degradation of MB, showing a degradation rate of up to 83% and 94% within 150 min for the pristine and Sn doped Bi_2_S_3_,  respectively.

**Conclusion:**

The enhancement in the photocatalytic activity of the Sn doped Bi_2_S_3_ was attributed to the suppression in the recombination rate of the electron‐hole pairs, due to the formation of new energy level below the CB, that was capable of altering the equilibrium concentration of the carrier. This confirmed that Sn doped Bi_2_S_3_ could be utilized as valuable cost-efficient catalysts for eliminating methyl blue from aqueous solutions and also possible candidates in environmental pollution treatment.

**Supplementary Information:**

The online version contains supplementary material available at 10.1186/s13065-021-00792-9.

## Introduction

Dyes are one of the harmful organic pollutants. They are released into the environment mainly from the textiles, foodstuffs, and leather industries [1, 2]. Their presence in effluents generally reduce sunlight transmission; thus, it affects photosynthesis, harms aquatic ecosystems and also the health and wellbeing of humans. Worse still, some of them are highly cytotoxic for the mammalian tissues [3]. Methylene blue (MB) is a basic dye, usually used for dyeing wool and silk. Like other dye materials, its removal from effluents is one of the major challenges in the industry. Different techniques, such as coagulation-flocculation processes, membrane filtration and ion exchange [4] have been developed to effect the remediation of water from toxic compounds. However, these methods are not suitable for industries that operate at a small scale due to high operational costs and sustainability. Therefore, techniques that are efficient, stable, and could operate at low cost continue to garner research interest.

Photo-enhanced degradation of pollutants by semiconductors is a new and effective technique for the removal of dyes from water. Under light irradiation, semiconductor-based photocatalysts could effectively decompose dyes. This occurs when the semiconductors are irradiated with photons whose energy is higher or equal to the photocatalyst’s band gap energy [5]. Bismuth sulfide (Bi_2_S_3_) is a typical environmentally friendly semiconductor photocatalyst. It is stable, has direct band gap energy of 1.30 eV, and possess a lamellar structure that makes it possible to be applied in photocatalysis [6, 7]. As an n-type semiconductor, it has great electron mobility and is applied in many areas, including supercapacitor electrodes, sensors, photodetectors, and thermoelectric devices, due to its reasonably low band gap energy [8, 9]. It has a great absorption coefficient in the range 104–105 cm^−1^, and a good incident photon to conversion efficiency of (∼5%) [10, 11]. It is possible to adjust the direct band gap energy by simply varying the size of the particles and shape, consequently resulting in some changes in the properties of the material. In addition, the band edges fall within the visible region of the solar energy spectrum. This makes it a very suitable material for devices that convert solar energy [12].

The introduction of dopants into semiconductor nanomaterials is significant since it impacts on the electrical, optical, catalytic, and magnetic properties of the host material [13]. It is also a strategy for improving the utilization of solar light for semiconductors, either by modifying the band gap structures or indirectly exchanging the energy. Band gap modification could be realized by creating impurity levels in the forbidden band of semiconductor or shifting the valence band edge. This method has been successfully employed to extend the light absorption range of some wide band gap semiconductors from UV to visible light, with appropriate metals or non-metal dopants [14]. In addition, the introduction of a suitable metal ion into a substrate such as a semiconductor host material suppresses the recombination of electron–hole pairs, thereby prolonging the lifetime of charge carriers. Consequently, the photocatalytic activity is improved efficiently and the apparent quantum efficiency can be enhanced [15].

Different transition metal-doped bismuth sulfide nanoparticles have been reported. Anasane and Ameta reported manganese (Mn^2+^) doped Bi_2_S_3_ nanoparticles of different morphologies [16]. A simple synthesis of Eu-doped Bi_2_S_3_ nanoparticles, in various ratios, through the breakdown of dual single source precursors: Bi(III) dithiocarboxylate and Eu(III) dithiocarboxylate complexes has been described [17, 18]. Microspheres of Sn-doped Bi_2_S_3_ (TDBs), composed of nanosheets consisting of Sn-doped Bi_2_S_3_ with a bit of Sn^4+^ substitution at Bi^3+^ sites within the Bi_2_S_3_ lattice has been reported [19]. Silver doped bismuth sulfide (AgBiS_2_) nanoparticles have been reported by single source precursor approach using bismuth diethyldithiocarbamate as the precursor under reflux condition in the presence of hexadecylamine [20]. The long chain amine acted as both the capping agent as well as shape directing coordinating solvent because it has been shown to control the rate of particle growth and size when used as a capping agent [21]. The good solar light absorbing potency of Bi_2_S_3_ has increased the research interest on its photocatalytic degradation of organic contaminants [22–24]. In this study, we present the synthesis, characterization and the photocatalytic degradation of methylene blue using undoped and Sn doped Bi_2_S_3_ nanomaterials.

## Experimental

### Materials

Analytical grade bismuth(III) chloride dihydrate, aniline, sodium hydroxide, carbon disulfide and ethanol were purchased from Merck. All reagents and solvents were analytical grade and used as received without further purification.

### Physical measurements

The infrared spectral studies of the complex were carried out using a Fourier-transform infrared (FTIR) spectrometer (Nicolet 560), in the wavenumber range of 4000–500 cm^−1^ at room temperature. The Nuclear Magnetic Resonance (NMR) spectra were recorded using a 600 MHz Bruker Avance III NMR spectrometer for ^1^H and ^13^C NMR analyses. The melting point measurement was done using a Gallenkamp melting point instrument. The absorption spectra of the bismuth sulfide nanoparticles were obtained using a Varian UV–vis spectrophotometer. The photoluminescence (PL) spectra were measured using Perkin Elmer LS 45 Fluorimeter. Powder X–ray diffractogram (XRD) of the nanoparticles were recorded on a Bruker D8 Advanced XRD machine, equipped with a proportional counter using Cu Kα radiation (λ = 1.5405 A, nickel filter). Samples were added on a flat steel sample holder and scanned from 10 to 80 °C. The diffraction peaks at several values were matched with other recorded standards in JCPDS. The morphology of the nanoparticles was studied using transmission electron microscopy-TEM (Hitachi HF–2000 TEM at 200 kV and FEI Tecnai G^2^ Twin at 20 kV).

### Synthesis of sodium *N*-phenyldithiocarbamate

A solution of NaOH (0.8 g, 0.02 mol) in 10 mL of distilled water was prepared in a round bottom flask. To this solution, aniline (1.86 g, 0.02 mol) was added and the mixture was stirred at a low temperature range ≤ 4 °C. After about 5 min, carbon disulfide (1.21 mL, 0.02 mol) was added slowly to the mixture. The faint yellowish-white solid product, which formed, was then filtered, rinsed with small portions of ether, and recrystallized in acetone.

Yield-1.56 g, 84.78%; M.pt 134–135 °C; Selected FTIR, υ (cm^−1^): 1442 (C=N), 1281 (C_2_–N), 986 (C=S), 3009 (=CH), 3400 (–NH), 1526 δ (NH).

### Synthesis of bismuth(III) tris(*N*-phenyldithiocarbamate)

The complex was prepared by reacting an aqueous solution of the ligand with an aqueous solution of BiCl_3_∙2H_2_O in 3:1 mol ratio (ligand to metal salt). Then, the precipitate formed was filtered, washed thoroughly using a mixture of ethanol (50 mL) and water (150 mL). The pure complex was obtained by dissolving the crude product in chloroform, filtering to remove the by product and subjecting the filtrate to slow evaporation.

Yield-1.39 g, 85.93%; M.pt: 153–154 °C; Selected FTIR, υ (cm^−1^): 1485 (C=N), 1232 (C_2_–N), 1012 (C=S), 2790 (–CH), 3007 (=CH), 3197 (NH), 1524 δ(–NH); ^1^H NMR (CDCl_3_) δ (ppm) = 7.91 (s, 3H, HN–C_6_H_5_), 7.35–7.18 (m, 15H, N-C_6_H_5_); ^13^C NMR (CDCl_3_) δ (ppm) = 179.97 (–NCS_2_), 137.13, 127.04, 129.56, 125.22 (HN-C_6_H_5_); Anal. calc.C_21_H_18_N_3_S_6_Bi (713.76): C, 35.34; H, 2.54; N, 5.89; S, 26.95; Found: C, 35.23; H, 2.14; N, 6.01; S, 26.34.

### Synthesis of pristine Bi_2_S_3_

In a typical procedure, 0.25 g of the bismuth complex was dispersed in 4 g oleylamine (OLA) in a 250 mL round-bottomed flask. This suspension was degassed for about 30 min, and then heated up to 180 °C under nitrogen, and maintained for 1 h. Thereafter, the reaction was terminated and the solution was allowed to cool down to 65 °C. Excess methanol was then added in order to flocculate the OLA capped bismuth sulfide nanorods. The product was isolated by centrifuging, rinsed 3 times with methanol and allowed to dry.

### Synthesis of Sn doped Bi_2_S_3_

Sn(IV) doped Bi_2_S_3_ were prepared by introducing Sn(IV) chloride (0.037 g) into a beaker containing the bismuth dithiocarbamate complex (0.25 g) and oleylamine (7.5 mL). The obtained solution was transferred into a 100 mL capacity Teflon-lined autoclave sample reactor vessel, and heated up to 180 °C. The reaction was maintained for 8 h. Thereafter, the solution was allowed to cool to room temperature, ethanol was added and the precipitate obtained was centrifuged and washed with ethanol. The precipitation process with ethanol and centrifuging was carried out three times and the samples were dried in air.

### Photocatalytic evaluation of the Bi_2_S_3_ and Sn doped Bi_2_S_3_

The photocatalytic activities of the Bi_2_S_3_ and Sn doped Bi_2_S_3_ were studied by measuring the degradation of methylene blue (30 mL, 10 mg/L) under visible light irradiation. 50 mg of the catalyst and dye solution, for each measurement, were mixed inside a Pyrex glass vessel. Then, the mixture was stirred in the dark cupboard for about 1 h to ensure that adsorption–desorption equilibrium was fully established between the dye and the catalyst. After 30 min, the solutions were exposed to the visible light with constant stirring in the compartment designed for the photocatalytic reaction. The efficiency of the degradation of the dye was analysed using UV–vis spectrometer by measuring the absorbance of aliquots taken at specific time intervals.

## Results and discussion

### Spectral studies

The FTIR spectrum of the ligand showed vibrational bands at 1479 cm^−1^ attributed to the stretching vibrations of C–N characteristic of dithiocarbamate compounds (Additional file 1). In the spectrum of the complex, a shift in the position of this band to around 1485 cm^−1^ occurred [25]. The position of this band was indicative of a partial double bond character of the C–N stretching vibrational band. The appearance of a single symmetric peak for the (C–S) stretching vibration around 1000 indicated a bidentate coordination fashion between the metal and the sulfur atoms of the dithiocarbamate ligand [26]. The stretching vibrations around 3009 cm^−1^ in the ligand was ascribed to the (=C–H) of the aromatic ring and these bands shifted upon complexation to a lower frequency, 3007 cm^−1^. The stretching vibrational band of N–H bond, observed at 3400 cm^−1^ in the spectrum of the ligand, shifted to 3197 cm^−1^ in the complex.

The ^1^H-NMR spectra of the complex (Additional file 1) showed the protons of the aromatic ring as multiplet in the range 7.10–7.35 ppm. The proton of the -NH group resonated at 7.91 ppm due to its attachment to nitrogen atom, similar to previous report [27]. In the ^13^C NMR spectra, the complexes showed carbon signal at 179 ppm, ascribed to the carbon of the thioureide bond. The appearance and position of this peak show the contribution of the double bond character in the complexes to a formally single N–C bond of the dithiocarbamate moiety [28]. The phenyl carbons from the dithiocarbamate moiety were affected by the coordination of the bismuth metal. Coordination of the bismuth atom to the respective ligands caused a decrease in the partial double bond character of the nitrogen–carbon bond as well as the movement of electron density towards the nitrogen from the carbon atom of the dithiocarbamate group. The additional deshielding of carbon sites upon complexation is a consequence of this electron movement [27].

### XRD studies of the nanomaterials

**Fig. 1:**
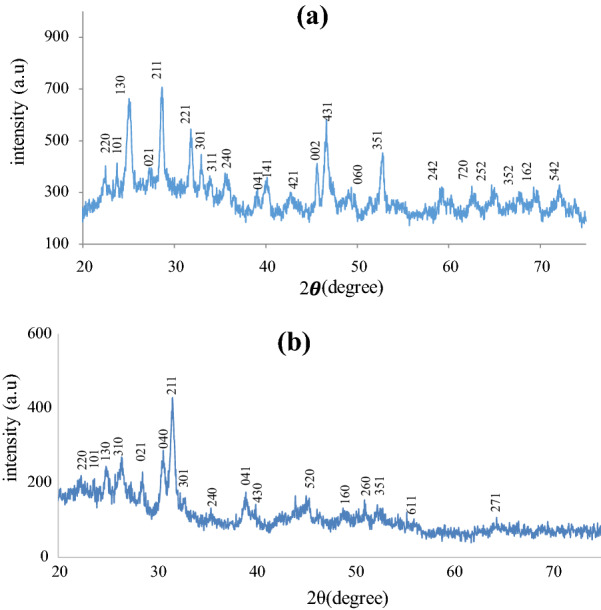
XRD of **a** undoped and **b** Sn doped Bi_2_S_3_nanomaterials obtained from bismuth(III) tris(N-phenyldithiocarbamate)

Figure 1a and b present the X-ray pattern of the undoped and Sn doped Bi_2_S_3_. Notable peaks observed at 2θ values of 23.39°, 24.92°, 28.60° and 31.79° could be indexed to the reflection pattern of (220), (130), (211) and (221) respectively of orthorhombic crystal structure of Bi_2_S_3_ with a JCPD No. 17-0320 [12]. Bi_2_S_3_ tends to crystalize in an orthorhombic system, with Pcnm space group, as it contains a thin layer arrangement of infinite chains of alternating Bi^3+^ and S^2−^. The intensity of the diffraction peaks of the Sn doped Bi_2_S_3_ are lower than that of the undoped Bi_2_S_3_. In addition, the diffraction peaks of the Sn doped Bi_2_S_3_ tend to be mildly displaced toward the zero mark of the 2θ value, indicating the doping of Sn into the lattice of Bi_2_S_3_.

### TEM analysis

**Fig. 2:**
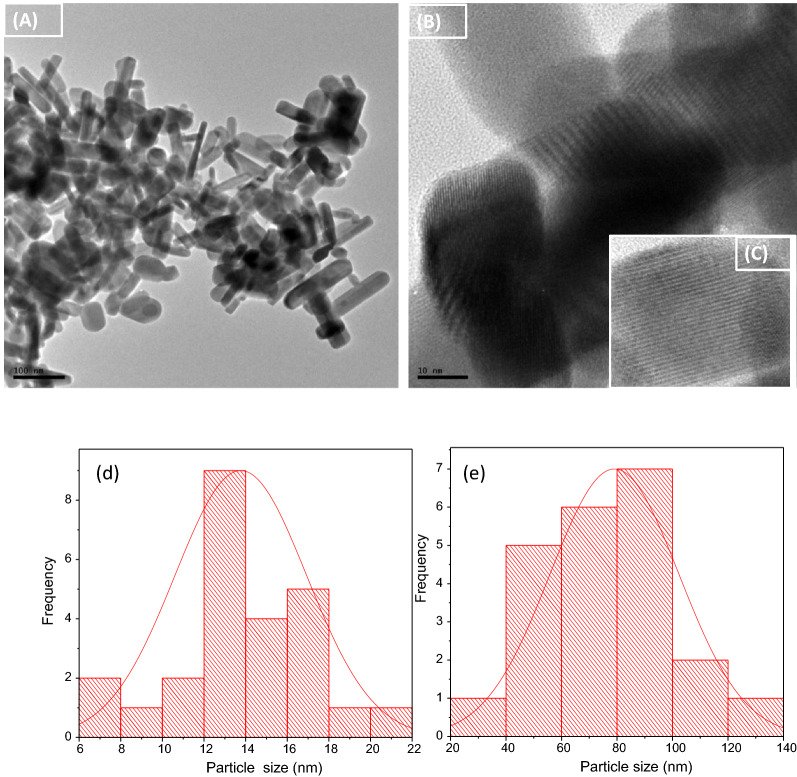
TEM micrograph of Bi_2_S_3_(2) nanorods at **a** low magnification, **b** high magnification, (c) inset-HRTEM showing lattice fringes; with corresponding particle size histogram showing (d) width and (e) length of the nanorods

The method of synthesis of nanomaterials influences the morphology and size. Figures 2a and b show low and high magnification images of the TEM micrograph of the Bi_2_S_3_, which displayed rod morphology. The inset in Fig. 2c shows clearly the lattice fringes of Bi_2_S_3_, which confirmed the crystalline nature of the nanorods. The particles size distribution histograms in Fig. 2d and e present the length and width of the nanorods, with average length and width size of 85.39 and 13.4 nm, respectively. The calculated aspect ratio for the nanorods was 6.3 nm, and they were fairly monodispersed with less than 40 percent superimposition on one another. The rod-like morphology exhibited by the Bi_2_S_3_ is partially due to the chain type structure of Bi_2_S_3_ nanoparticles, which is known to form band structures as Bi units interlinked with weaker van der Waals forces [29].

**Fig. 3:**
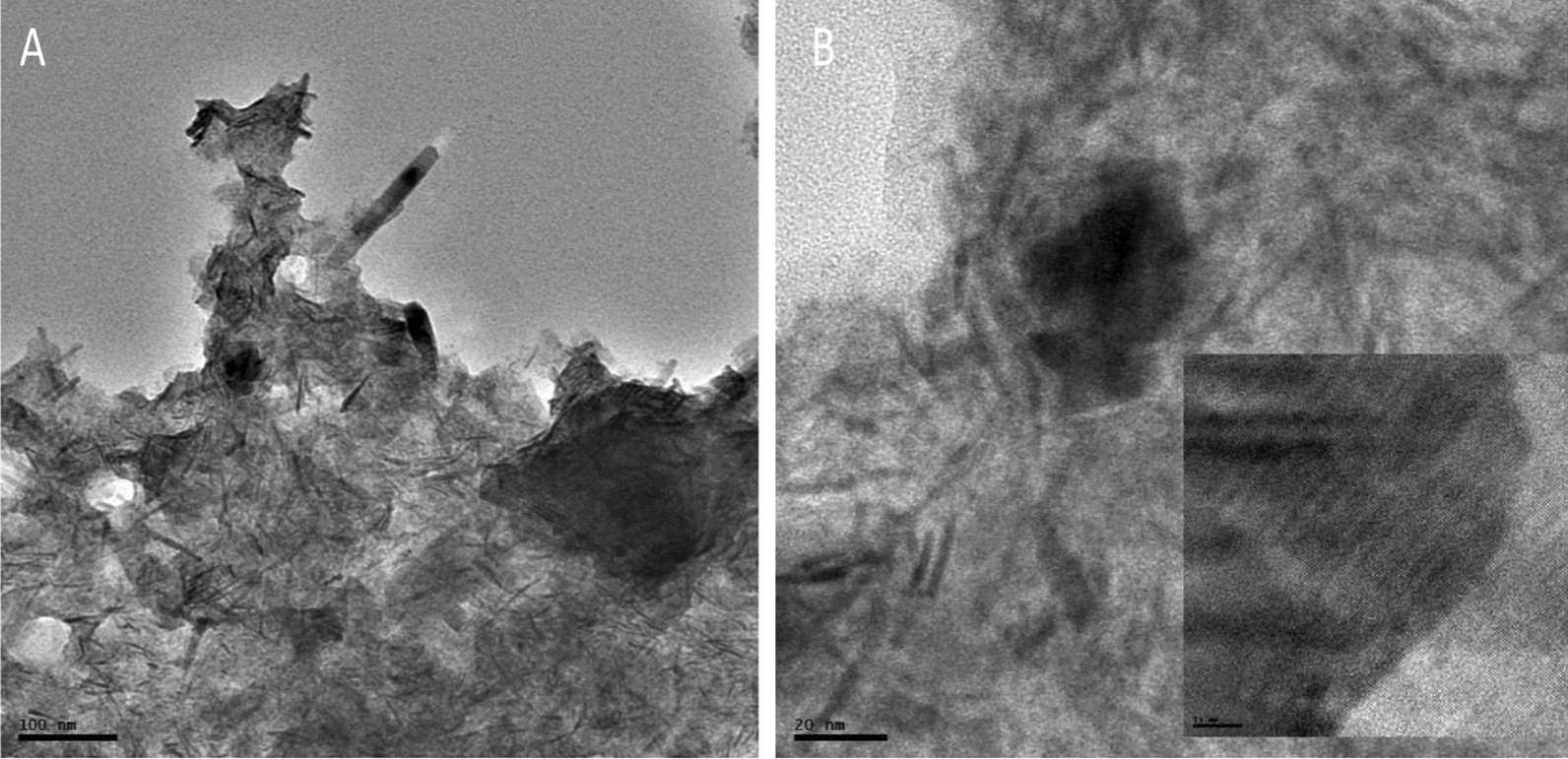
TEM images of Sn doped Bi_2_S_3_(2) at **a** low magnification, **b** high magnification, inset-HRTEM showing lattice fringes

**Fig. 4:**
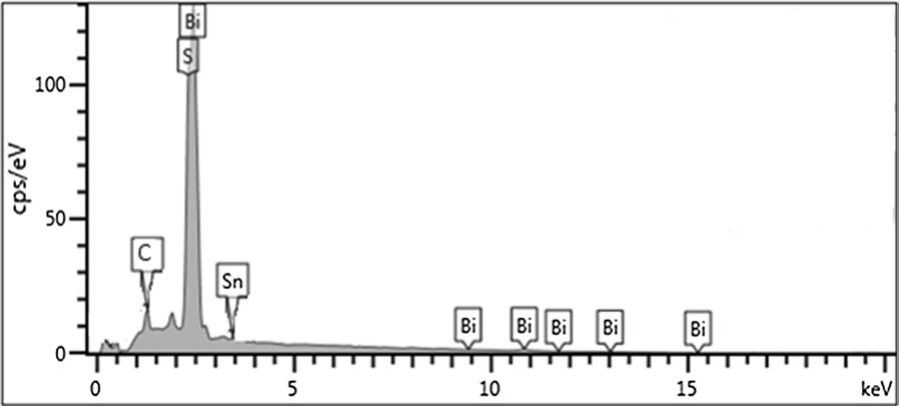
EDX spectrum of Sn doped Bi_2_S_3_ prepared from bismuth(III) tris(N-phenyldithiocarbamate)

The micrograph of the Sn doped Bi_2_S_3_ shown in Fig. 3a and b, which appeared as needle-like structures covered by semi-transparent creased and wrinkle materials layers. The dimension of these needles was measured in terms of length and width. The observed average size of the width was 2.19 nm, while the length was 83.29 nm. Inset is the HRTEM that shows lattice fringes which indicated the crystallinity of the nanomaterial. The EDX spectrum presented in Fig. 4 shows peaks of Sn, Bi, and S which confirmed the formation of bismuth sulfide and a successful doping of the nanomaterial with Sn. The carbon peak could be attributed to the carbon tape used to hold the samples firmly to the stud during analysis.

### Photocatalytic degradation of methylene blue

The wavelength of maximum absorption of methylene blue (MB) is 664 nm, which is due to the absorption of the π-system [30]. MB could be degraded either by oxidative degradation of its molecule or by a two-electron reduction [31, 32]. In the presence of a semiconductor such as Bi_2_S_3_, the degradation of MB is not a consequence of a direct redox reaction between the Bi_2_S_3_ and the dye molecules but results from the interaction of the dye molecules with radicals. The pathway for the photocatalytic degradation of MB in water has been proposed [33]. During photo-enhanced degradation, the electrons generated in the Bi_2_S_3_, under the irradiation of visible light, would either recombine with the holes of the valence band or react with the adsorbed oxygen on the surface of Bi_2_S_3_. In the latter case, oxygen ions are created, which in turn combine with the adsorbed water molecules to form hydroxyl radicals ^•^OH. Eventually, the reactions of MB with the radicals cause the degradation of the dye molecules. The sequence of this process could be itemized as follows:

In the first equation, the activation of Bi_2_S_3_ by incident light (hυ) generates excitons (electron–hole pairs), which are powerful oxidizing and reducing agents, respectively [34]:

1 $${\text{Bi}}_{{2}} {\text{S}}_{{3}} + hv \to e^{ - } + h^{ + }$$

This is followed with the oxidation of the adsorbed water molecules on the surface of Bi_2_S_3_, which then results into the production of hydroxyl radical according to reaction:

2 $${\text{H}}_{{2}} {\text{O}} \to {\text{H}}^{ + } +^{ \cdot}{\text{OH}}$$

This process is followed with the reduction and oxidation reactions as follows:

3 $${\text{Reduction}} - {\text{ OH}}^{ - } + h^{ + } \to^{ \cdot}{\text{OH}}$$

4 $${\text{Oxidation}} - {\text{ O}}_{{2}} + e^{ - } \to^{ \cdot}{\text{O}}_{{2}}^{-}$$

Finally, the degradation of the organic compound (MB) occurs:

5 $${\text{MB}} +^{ \bullet } {\text{OH}} \to {\text{products }}\left( {{\text{CO}}_{{2}} + {\text{ H}}_{{2}} {\text{O }} + {\text{ NH}}_{{4}}^{ + } + {\text{NO}}_{{3}}^{ - } + {\text{ SO}}_{{4}}^{{{2} - }} + {\text{Cl}}^{ - } } \right)$$

**Fig. 5:**
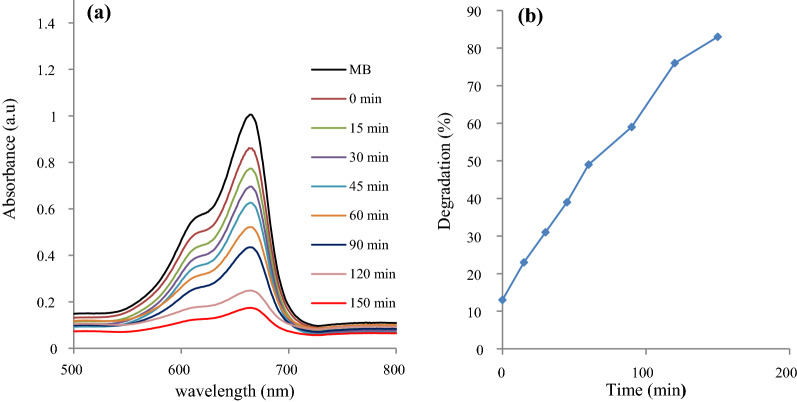
**a** Absorption spectra of aqueous MB at different time intervals, and **b** percentage degradation of MB with change in time using Bi_2_S_3_

**Fig. 6:**
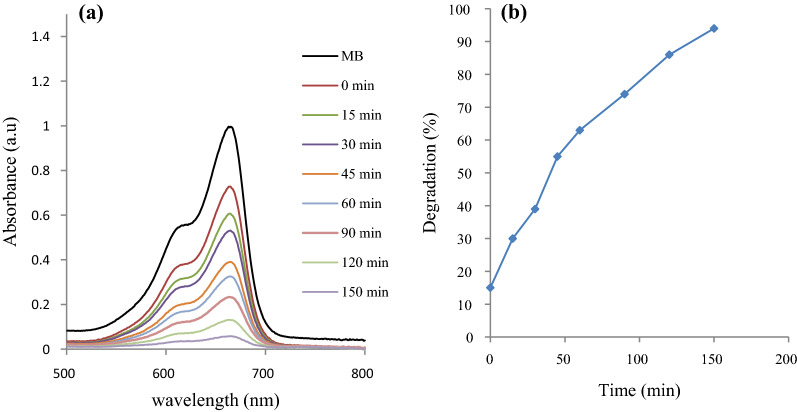
**a** Absorption spectra of aqueous MB at different time intervals, **b** percentage degradation of MB using Sn doped Bi_2_S_3_

The photodegradation efficiency of the synthesized Bi_2_S_3_ on the MB is shown in Fig. 5, while that of the Sn doped Bi_2_S_3_ is presented in Fig. 6. The UV–vis spectra of the original and degraded MB solutions were determined by a UV–vis spectrophotometer at 15 min time interval and over a 150 min period. The Figures showed pronounced reduction with time in the absorption peaks of the MB solutions at 664 nm in the presence of the nanoparticles, due to an oxidative degradation process [9], suggesting a breakdown of the chromophore in the reaction process. The photocatalytic activity of Bi_2_S_3_ photocatalysts for MB photodegradation was calculated using the equation:

6 $${\text{Degradation \% = }}\frac{{A_{c} - A_{t} }}{{A_{c} }} \times 100$$

where A_*c*_ is the initial solution of dye concentration and A_*t*_ is the final solution of dye concentration after treating with the photocatalyst.

The efficiency of the pristine Bi_2_S_3_ for the dye degradation was 83%, and increased to about 94% with the Sn doped Bi_2_S_3_ samples. Considerable improvement in the photocatalytic activity of doped semiconductors in aqueous systems has been reported for the degradation of organic compounds [35–37]. The electronic structure in a semiconductor could be effectively modulated by the introduction of a dopant into its lattice. Since the ionic radius of Sn is smaller than that of Bi, the replacement of Bi atoms by Sn atom is promoted. The introduction of Sn could form a new energy level below the CB, which could alter the equilibrium concentration of the carrier. The new energy level serves as electron‐hole trapping site. Hence, electron transfer to the Sn causes a reduction in the carrier recombination rate, and allows an effective reaction between the electrons and the surface trapped holes. This is capable of suppressing the recombination rate of electron‐hole pairs, and consequently enhances the degradation rates. This accounts for the improvement in the photocatalytic performance of the doped Bi_2_S_3_ compared with the pristine Bi_2_S_3_.

**Fig. 7:**
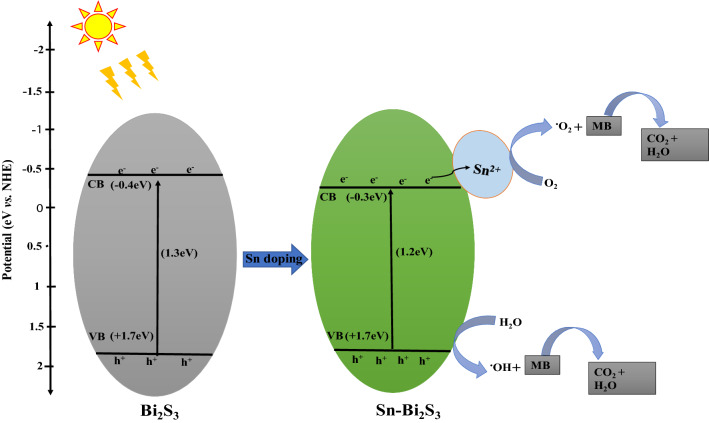
Schematic representation of degradation of MB in **a** pure Bi_2_S_3_, **b** metal-doped Bi_2_S_3_ with intermediate band

The degradation process, showing the energy diagram with the different band level positions are shown in Fig. 7. The doped Sn could promote the separation of photo-excited electron–hole pairs effectively. thereby suppressing the recombination of electron–hole. Consequently, the photocatalytic degradation activity is enhanced by the doped Sn. Table 1 presents a summary of the efficiency of different metal and non-metal doped Bi_2_S_3_ in comparison with the results of the current study.

**Table 1:** The efficiency of different metal and non-metal doped Bi_2_S_3_ for the photocatalytic degradation of dyes

Compounds	Synthesis method	Morphology	Pollutants (dye)	Degradation (%)	Refs.
Sn-Bi_2_S_3_	Hydrothermal	Nanorods	MB	83–94	This work
Se-Bi_2_S_3_	Hydrothermal	Nanords	MB	88–96	[38]
Au-Bi_2_S_3_	Facile one-pot	Nanorods	MO	72–80	[39]
		Nanoflowers	MO	86–97	
		Nanorods	RhB	77	
		Nanoflowers	RhB	95	
Sn-Bi_2_S_3_	Solvothermal	Microspheres	RhB	21–99	[40]
Mn-Bi_2_S_3_	Wet chemical	Nanorods	MV	75–90	[16]
Eu-Bi_2_S_3_	Solvothermal	Nanoflowers	MB	4.6	[17]

## Conclusion

Orthorhombic Bi_2_S_3_ and doped Bi_2_S_3_ nanostructures have been prepared using a single source precursor complex in oleylamine. Both the diameter and length of the nanorods was observed to be decreased with doping with tin. The photocatalytic evaluation of the Bi_2_S_3_ and doped Bi_2_S_3_ nanomaterials showed good photocatalytic activity for the degradation of methylene blue. The doped Bi_2_S_3_ had higher degradation efficiency due to them having a new energy level, which can suppress the recombination rate of electron‐hole pairs. The high photocatalytic activities is suggestive that the reported Bi_2_S_3_ nanostructures would be good candidates in the photocatalytic degradation of organic pollutants in aqueous solution.

## Supplementary Information

**Additional file 1: Fig. S1.** FTIR spectra of (a) sodium N-phenyldithiocarbamate and (b) bismuth(III) tris(N-phenyldithiocarbamate). **Fig. S2.** (a)^1^H and (b)^13^C NMR spectra of Bi(III) tris (N-phenyldithiocarbamate) complex.

## Data Availability

Available on request. Contact the corresponding author.

## References

[1] Bhatnagar A, Jain AK (2005). A comparative adsorption study with different industrial wastes as adsorbents for the removal of cationic dyes from water. J Colloid Interface Sci.

[2] Robinson T, McMullan G, Marchant R, Nigam P (2001). A comparative adsorption study with different industrial wastes as adsorbents for the removal of cationic dyes from water. Bioresour Technol.

[3] Azizullah A, Khattak MN, Richter P, Hader DP (2011). Water pollution in Pakistan and its impact on public health-a review. Environ Int.

[4] Raghu S, Ahmed Basha C (2007). Chemical or electrochemical techniques, followed by ion exchange, for recycle of textile dye wastewater. J Hazard Mater.

[5] Park JS, Choi W (2004). Enhanced remote photocatalytic oxidation on surface-fluorinated TiO_2_. Langmuir.

[6] Ye C, Meng G, Jiang Z, Wang Y, Zhang L (2002). Rational growth of Bi_2_S_3_ nanotubes from quasi-two-dimensional precursors. Chem Soc.

[7] Sun B, Qiao Z, Shang K, Fan H, Ai S (2013). Facile synthesis of silver sulfide/bismuth sulfide nanocomposites for photocatalytic inactivation of *Escherichia coli* under solar light irradiation. Mater Lett.

[8] Konstantatos G, Levina L, Tang J, Sargent EH (2008). Sensitive solution-processed Bi_2_S_3_ nanocrystalline photodetectors. Nano Lett.

[9] Helal A, Harraz FA, Ismail AA, Sami TM, Ibrahim IA (2016). Controlled synthesis of bismuth sulfide nanorods by hydrothermal method and their photocatalytic activity. Mater Des.

[10] Dutta SK, Mehetor SK, Pradhan N (2015). Metal semiconductor heterostructures for photocatalytic conversion of light energy. J Phys Chem Lett.

[11] Saha N, Sarkar A, Ghosh AB, Dutta AK, Bhadu GR, Paul P, Adhikary B (2015). Highly active spherical amorphous MoS_2_: facile synthesis and application in photocatalytic degradation of rose bengal dye and hydrogenation of nitroarenes. RSC Adv.

[12] Pejova B, Grozdanov I (2006). Structural and optical properties of chemically deposited thin films of quantum-sized bismuth(III) sulfide. Mater Chem Phys.

[13] Manzoor M, Rafiq A, Ikram M, Nafees M, Ali S (2018). Structural, optical, and magnetic study of Ni-doped TiO_2_ nanoparticles synthesized by sol-gel method. Intern Nano Lett.

[14] Anku WW, Oppong SOB, Govender PP. Bismuth-based nanoparticles as photocatalytic materials. In Bismuth -Adv. Appl. Defects Charact. 2018.

[15] Dixit N, Anasane N, Chavda M, Bodas D, Soni HP (2013). Inducing multiple functionalities in ZnS nanoparticles by doping Ni^2+^ ions. Mater Res Bull.

[16] Anasane N, Ameta R (2017). Morphologies of nanostructured bismuth sulphide and Mn (II) doped bismuth sulphide nanoparticles: characterization and application. Mater Sci Pol.

[17] Sarkar A, Ghosh AB, Saha N, Dutta AK, Srivastava DN, Paul P, Adhikary B (2015). Enhanced photocatalytic activity of Eu-doped Bi2S3 nanoflowers for degradation of organic pollutants under visible light illumination. Catal Sci Technol.

[18] Dutta AK, Maji SK, Mitra K, Sarkar A, Saha N, Ghosh AB, Adhikary B (2014). Single source precursor approach to the synthesis of Bi2S3 nanoparticles: a new amperometric hydrogen peroxide biosensor. Sens Actuators B Chem.

[19] Rong X, Qiu F, Rong J, Yan J, Zhao H, Zhu X, Yang D (2015). Synthesis of porous g-C_3_N_4_/La and enhanced photocatalytic activity for the degradation of phenol under visible light irradiation. J Solid State Chem.

[20] Yu Y-Q, Zhang B-P, Ge Z-H, Shang P-P, Chen Y-X (2011). Thermoelectric properties of Ag-doped bismuth sulfide polycrystals prepared by mechanical alloying and spark plasma sintering. Mater Chem Phys.

[21] Soofivand F, Salavati-Niasari M, Mohandes F (2013). Novel precursor-assisted synthesis and characterization of zinc oxide nanoparticles/nanofibers. Mater Lett.

[22] Kim J, Kang M (2012). High photocatalytic hydrogen production over the band gap-tuned urchin-like Bi_2_S_3_-loaded TiO_2_ composites system. Int J Hydrog Energ.

[23] Kumar S, Sharma S, Sood S, Umar A, Kansal SK (2016). Bismuth sulfide (Bi_2_S_3_) nanotubes decorated TiO2 nanoparticles heterojunction assembly for enhanced solar light driven photocatalytic activity. Ceram Intern.

[24] Lu J, Han Q, Wang Z (2012). Synthesis of TiO_2_/Bi_2_S_3_ heterojunction with a nuclear-shell structure and its high photocatalytic activity. Mater Res Bull.

[25] Abdullah NH, Zainal Z, Silong S, Tahir MIM, Tan KB, Chang SK (2016). Synthesis of zinc sulphide nanoparticles from thermal decomposition of zinc N-ethyl cyclohexyl dithiocarbamate complex. Mater Res Bull.

[26] Ajibade PA, Onwudiwe DC, Moloto MJ (2011). Synthesis of hexadecylamine capped nanoparticles using group 12 complexes of N-alkyl-N-phenyl dithiocarbamate as single-source precursors. Polyhedron.

[27] Bobinihi FF, Osuntokun J, Onwudiwe DC (2018). Syntheses and characterization of nickel(II) dithiocarbamate complexes containing NiS_4_ and NiS_2_PN moieties: nickel sulphide nanoparticles from a single source precursor. J Saudi Chem Soc.

[28] Srinivasan N, Thirumaran S, Ciattini S (2009). Effect of position of methyl substituent in piperidinedithiocarbamate on the ZnS4N chromophore: synthesis, spectral, valence-bond parameters and single crystal X-ray structural studies on bis(2-methylpiperidinecarbodithioato-S, S′)-(pyridine)zinc(II) and bis(4-methylpiperidinecarbodithioato-S, S′)(pyridine)zinc(II). J Mol Struct.

[29] O'Brien P, Malik MA, Revaprasadu N (2007). Precursor routes to semiconductor quantum dots. Phosphorus Sulfur Silicon Relat Elem.

[30] Rache ML, García AR, Zea HR, Silva AMT, Madeira LM, Ramírez JH (2014). Azo-dye orange II degradation by the heterogeneous Fenton-like process using a zeolite Y-Fe catalyst-kinetics with a model based on the Fermi's equation. Appl Catal B Environ.

[31] Mahmoud MHH, Ismail AA, Sanad MMS (2012). Developing a cost-effective synthesis of active iron oxide doped titania photocatalysts loaded with palladium, platinum or silver nanoparticles. Chem Eng J.

[32] Fateh R, Ismail AA, Dillert R, Bahnemann DW (2011). Highly active crystalline mesoporous TiO_2_ films coated onto polycarbonate substrates for self-cleaning applications. J Phys Chem C.

[33] Houas A, Lachheb H, Ksibi M, Elaloui E, Guillard C, Herrmann JM (2001). Photocatalytic degradation pathway of methylene blue in water. Appl Catal B: Environ.

[34] Mehrabian M, Esteki Z (2017). Degradation of methylene blue by photocatalysis of copper assisted ZnS nanoparticle thin films. Optik.

[35] Yan J, Zhang Y, Liu S, Wu G, Li L, Guan N (2015). Facile synthesis of an iron doped rutile TiO_2_ photocatalyst for enhanced visible-light-driven water oxidation. J Mater Des A.

[36] Li X, Guo Z, He T (2013). The doping mechanism of Cr into TiO_2_ and its influence on the photocatalytic performance. Phys Chem Chem Phys.

[37] Archana PS, Jose R, Jin TM, Vijila C, Yusoff MM, Ramakrishna S (2010). Structural and electrical properties of Nb-doped anatase TiO_2_ nanowires by electrospinning. J Am Ceram Soc.

[38] Song L, Chen C, Zhang S (2011). Preparation and photocatalytic activity of visible light-sensitive selenium-doped bismuth sulfide. Powder Tech.

[39] Nwaji N, Akinoglu EM, Giersig M (2021). Gold nanoparticle-decorated Bi_2_S_3_ nanorods and nanoflowers for photocatalytic wastewater treatment. Catalysts.

[40] Jiang Y, Hu J, Li J (2016). Synthesis and visible light responsed photocatalytic activity of Sn doped Bi_2_S_3_ microspheres assembled by nanosheets. RSC Adv.

