# Supplementary material for: Solvothermal synthesis of pure and Sn-doped Bi2S3 and the evaluation of their photocatalytic activity on the degradation of methylene blue
Source: BMC Chem. 2021 Dec 18;15(1):65. doi: 10.1186/s13065-021-00792-9 (PMC8684666; doi:10.1186/s13065-021-00792-9)
Supplement: Supplementary file 1 — Additional file 1: Fig. S1. FTIR spectra of (a) sodium N-phenyldithiocarbamate and (b) bismuth(III) tris(N-phenyldithiocarbamate). Fig. S2. (a)1H and (b)13C NMR spectra of Bi(III) tris (N-phenyldithiocarbamate) complex. [file 13065_2021_792_MOESM1_ESM.docx]

Additional materials

**(b)**

**(a)**

**Figure S1:** FTIR spectra of (a) sodium *N*-phenyldithiocarbamate and (b) bismuth(III) tris(*N*-phenyldithiocarbamate)

| **(a)**  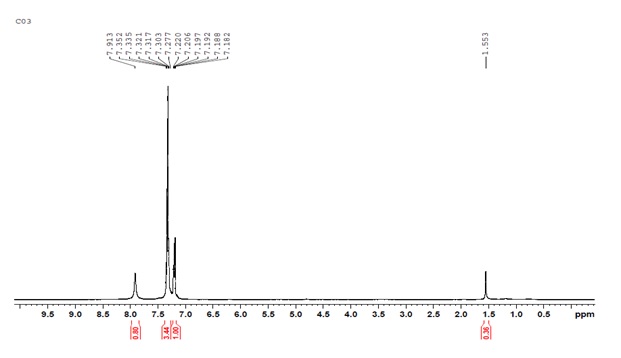 |
| --- |
| **(b)** |
| 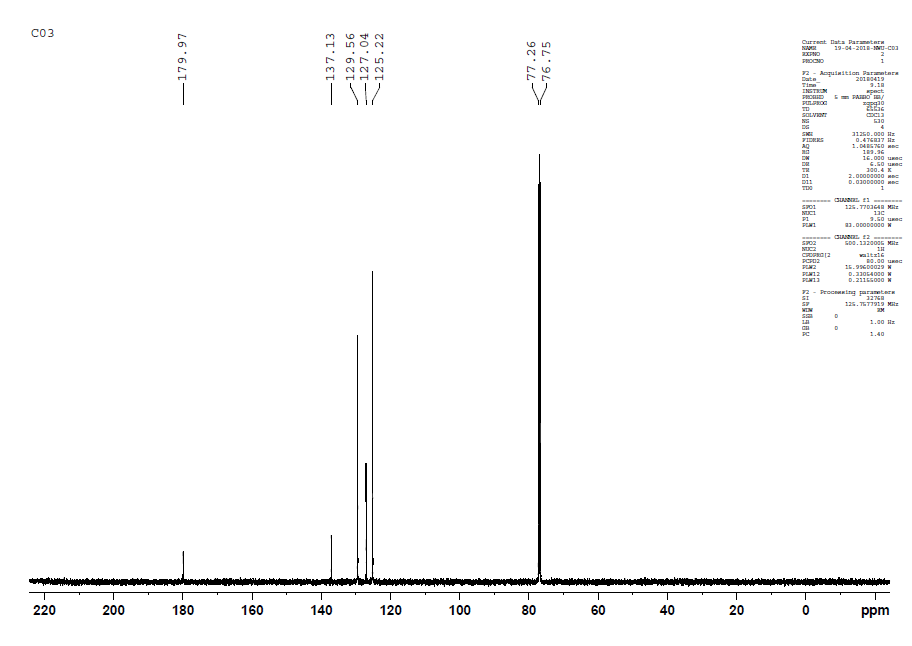   |

**Figure S2**: **(a)**^1^H and **(b)**^13^C NMR spectra of Bi(III) tris (N-phenyldithiocarbamate) complex
